# The Potential of Plant-Based Lifestyle Interventions to Reduce the Burden of Disease in a Multi-Crisis Era

**DOI:** 10.1177/15598276261418594

**Published:** 2026-01-28

**Authors:** Komathi Kolandai, Nicholas Wright, Luke Wilson, Heleen Haitjema, Summer Rangimaarie Wright, Meika Foster, George Laking, Marissa Kelaher, Reen Skaria, Jennifer Douglas, Mok Keong Liew, Marion Leighton, Deborah Brunt, Fuchsia Gold-Smith, Mark Craig, Thomas Joseph, Wayne Hurlow, Cheryl Pittar, Sarah Mortimer

**Affiliations:** 1 COMPASS Research Centre & Public Policy Institute, University of Auckland, Auckland, New Zealand (KK); 2 Royal New Zealand College of GPs, Wellington, New Zealand (NW); 3 Doctors for Nutrition / Miramar Medical Centre, Wellington, New Zealand (LW); 4 Doctors for Nutrition, Adelaide, Australia (HH); 5 Edible Research Ltd / OraTaiao: Aotearoa NZ Climate & Health Council, New Zealand (SRW); 6 Edible Research Ltd, New Zealand (MF); 7 Centre for Cancer Research, University of Auckland, Auckland, New Zealand (GL); 8 Your Lifestyle Medics, Nelson, New Zealand (MK); 9 63589 School of Nursing, Southern Institute of Technology, Invercargill, New Zealand (RS); 10 Jump Start Nutrition, Dunedin, New Zealand (JD); 11 Greenlane Medical Specialists, Auckland, New Zealand (MKL); 12 Greenstone Consultants, Wellington, New Zealand (ML); 13 Rebelle Health, Dunedin, New Zealand (DB); 14 Feed Nutrition, Auckland, New Zealand (FGD); 15 True South Medical, Ponsonby Medical Centre, Auckland, New Zealand (MC); 16 He Puna Waiora Wellness Centre, Invercargill, New Zealand (TJ); 17 Wakefield and Districts Community Health Centre, Nelson, New Zealand (WH); 18 Your Lifestyle Medics, Mount Maunganui, New Zealand (CP); 19 Seed Nutrition, Mount Maunganui, New Zealand (SM)

**Keywords:** lifestyle intervention, preventive healthcare, plant-based diet, COVID-19, zoonotic diseases, non-communicable diseases, climate-change-induced diseases, healthcare policy

## Abstract

This transdisciplinary, evidence-based viewpoint draws attention to literature suggesting that formalized plant-based lifestyle interventions have the potential to reduce the risk of COVID-19 and non-communicable diseases. Such interventions also offer the health sector a way to contribute to mitigating the risk of new zoonotic diseases and reducing carbon emissions (and, consequently, climate-change-induced diseases), all of which would help lower the overall disease burden. However, several challenges must be addressed to incorporate plant-based lifestyle interventions into clinical medicine. These include generating more methodologically robust and convincing evidence on the COVID-19–diet link, enhancing physicians’ understanding of plant-based diets, and ensuring equitable access to affordable, culturally inclusive, nutritionally adequate, and appealing plant-based foods. Contextual barriers, such as counteraction from profit-driven industries, and personal barriers, such as psychological resistance, must also be acknowledged and mitigated. While not without obstacles, plant-based lifestyle interventions merit consideration given their multifaceted potential to enhance both human and planetary health.

“Doctors’ recommendations have the highest potential to encourage the adoption of plant-based diets, even among those reluctant to do so”

## Introduction

Prior to COVID-19, the growth of zoonotic and non-communicable diseases was already causing enormous burden on health sectors worldwide.^1,2^ The COVID-19 pandemic (caused by the SARS-CoV-2 coronavirus) increased this overall disease burden, elevated pressures on the health system and adversely affected the lives of health professionals.^3^ Globally, COVID-19 led to an enormous cost to society as measured by excess mortality, and treatment and mitigation expenses.^4^ The production, testing, and distribution of novel COVID-19 vaccines also entailed significant costs.^5^ The effectiveness of these vaccines rapidly waned over time for new virus variants^6^—suggesting an ongoing manufacturing cost in vaccine reliance.

Post-acute sequelae of COVID-19 or long-Covid present the risk of multiple morbidities including neurological, pulmonary, renal, musculoskeletal, cardiac, and gastrointestinal conditions, which entail direct medical costs experienced at the individual level and long-term costs to society, further increasing the overall burden of disease.^7,8^ The pandemic also entailed indirect socioeconomic costs such as lockdowns leading to mental health decline, unemployment, adverse effects on children’s learning, and increased medical waste leading to detrimental environmental effects.^9^

While the origin of SARS-CoV-2 remains inconclusive, zoonotic disease trends suggest the next global pandemic is only a matter of time^10^ alerting that society will face this costly multipronged burden again, which, in turn, questions the logic of maintaining a reactive model of responding to zoonotic diseases predominantly through tertiary prevention and acute care.

This transdisciplinary viewpoint aims to encourage discussion, further exploration, and consideration of plant-based diets as a lifestyle intervention, given the public health relevance and need for modifiable risk factors in disease prevention. A plant-based diet encompasses vegan, vegetarian and semi-vegetarian dietary patterns emphasizing the consumption of predominantly plant-based foods with reduced or no animal products, though many researchers have used the term interchangeably with a vegan diet.^11,12^ Plant-based diets may act as a protective factor against diseases through several possible mechanisms. For instance, being rich in phytochemicals (e.g., carotenoids, glucosinolates, flavonoids), vitamins (e.g., C and E), and fiber, and lower in saturated fats, they offer antioxidation, anti-inflammatory, and immunity enhancement functions.^13,14^ Fiber, found exclusively in plants, diversify and stabilize gut microbiota to help maintain essential intestinal functions, blood-brain barrier integrity, and enhance resistance to pathogens.^15^

In the United States (US), some hospitals focusing on a preventive approach have begun implementing plant-based programmes for staff and patients to promote healthy eating, prevent chronic diseases, support recovery from illnesses, and prevent illness recurrence while using their purchasing power to promote healthier food options.^16,17^ In Germany and the United Kingdom (UK), hospitals have explored ways to introduce plant-based food programmes to reduce environmental impact and carbon footprint in addition to promoting health.^18,19^ However, plant-based lifestyle interventions remain underemphasized in most health sectors globally.

In the following sections, we note emerging, albeit limited, research suggesting that plant-based lifestyle interventions could potentially reduce the risk of infection and severity of COVID-19 (i.e., more serious disease and hospital admission). We also briefly discuss the value of plant-based diets in mitigating new zoonotic diseases, non-communicable diseases, and climate-change-induced diseases. We end with a discussion of the implications of plant-based lifestyle interventions for health sectors.

## COVID-19 Infection and Severity

The COVID-19 pandemic prompted researchers to investigate the influence of dietary patterns on COVID-19 severity and clinical outcomes. Supplementary Document 1 details findings from several of these studies involving health-care workers, COVID-19 patients, and the general population, which we considered in the preparation of the present Viewpoint.

A systematic review of thirteen observational studies on diet quality and COVID-19 risk found that adherence to a healthy plant-based diet was associated with lower COVID-19 infection and severity in a subgroup analysis.^20^ Subgroup analyses from a systematic review and meta-analysis of 5 studies involving over 4 million participants indicated that plant-based diets were associated with a 50% reduction in COVID-19 infection risk, whereas Mediterranean diets were linked to a 22% reduction.^21^ Similarly, a systematic meta-analysis of 7 observational studies found that higher adherence to plant-based diets was consistently associated with a lower risk of COVID-19 infection and hospitalization, suggesting a potential protective role of plant-based dietary patterns against both infection and severe outcomes.^22^

Similar observations regarding the benefits of plant-based diets in minimizing COVID-19 severity, hospitalization and even long-COVID-related symptoms and mortality were reported in 6 other reviews.^13,23-27^ In some cases, plant-based diets were considered “beneficial in directly reducing the risk of severe COVID-19 symptoms and the risk of infection” independently of other health factors.^23^

However, studies examining the effects of plant-based diets on COVID-19 outcomes remain limited in both number and methodological rigor and largely exploratory. Given that these studies vary in terms of the type of plant-based diet category examined, it remains unclear which category is most effective for reducing COVID-19 risk. Furthermore, the challenging context of the pandemic restricted most studies to observational designs, and, in some cases, small sample sizes, with incomplete consideration of confounding factors (e.g., not all studies accounted for lifestyle factors or COVID-19 protective behaviors such as mask wearing, hand washing and social distancing). Vaccination status was also not considered in most studies, in part because many were conducted prior to vaccine roll out. Hence, based on the current body of research, a causal link between plant-based diets and COVID-19 outcomes cannot be established.

## New Zoonotic Diseases

According to the United Nations Environment Programme and preceding studies, increased demand for animal protein, unsustainable intensive agriculture, and wildlife trade are among the primary drivers of zoonotic diseases, which can lead to epidemics and pandemics.^10,28-33^ While it remains inconclusive whether SARS-CoV-2 was zoonotic, the COVID-19 pandemic prompted scholarly attention to the simple logic of shifting from meat-based to plant-based diets to prevent future pandemics—an approach offering likely the most effective primary prevention given that zoonotic transmissions are prevented at their source.^34-38^

## Non-communicable Diseases

There is substantial evidence, based on an established body of research, demonstrating the preventive benefits of plant-based diets against non-communicable diseases. A meta-analysis of 108 studies reported that vegetarians and vegans had significantly lower levels of body mass index, cholesterol, and fasting glucose (i.e., risk factors for chronic diseases) compared to nonvegetarians and nonvegans.^39^ A systematic review of 44 nutrition intervention studies (half of which excluded animal products entirely) indicated that plant-based diets were associated with a range of health benefits such as weight management and improved cardiovascular health.^11^ Likewise, a systematic review of 32 longitudinal studies found a consistent pattern of health benefits from plant-based diets including enhanced metabolic function, effective weight control, decreased cardiovascular risks, and beneficial impacts on gut microbiota and inflammation levels.^40^ A systematic review and meta-analysis of 18 studies reported that plant-based diets were associated with a 26% lower incidence of chronic kidney disease.^41^ Plant-based diets also reduce blood pressure, the risk of obesity and related morbidities such as type 2 diabetes mellitus, the risk of heart disease and the risk of various forms of cancer.^11,39,40,42^

University of Oxford researchers, using a “cost-of-illness” approach to estimate the “direct health-care costs and the indirect costs of informal care and lost work days that are associated with deaths from specific diseases,” reported that relative to a reference scenario based on FAO projections, shifts to vegetarian and vegan diets can result in yearly savings of $973 and $1067 billion US respectively.^43^ A modeling study of the EAT-Lancet diet in New Zealand illustrated that this dietary approach could generate a savings of 1.4 million quality adjusted life years and an approximate NZD 20 billion in health system cost savings.^44^

## Climate-Change-Induced Diseases and Health Risks

Growing evidence shows the favorable impacts of plant-based diets on not only human but also planetary health.^45-47^ Globally, 57% of food-related greenhouse gas emissions is attributed to animal-based food production, which is almost double the 29% attributed to plant-based foods.^48^ A meta-analysis of data from 38 700 commercial farms in 119 countries detailed how moving away from animal-based foods can lead to a 49% reduction in greenhouse gas emissions in addition to reductions in other environmental pressures such as land use, water use, eutrophication, and terrestrial acidification.^49^ Another study that considered the food-related greenhouse gas emission data from 54 high-income nations showed how shifting to the primarily plant-based EAT–Lancet planetary health diet could reduce the nations’ annual agricultural emissions by 61.5%.^50^ These reductions and associated climate change mitigations, have the potential to protect against multiple harmful health impacts, including emergent vector- food- and water-borne infectious diseases, adverse effects of heat stress, respiratory, cardiovascular and neurological effects, as well as climate-induced mental health decline.^51-55^

## Discussion

**Figure 1.**
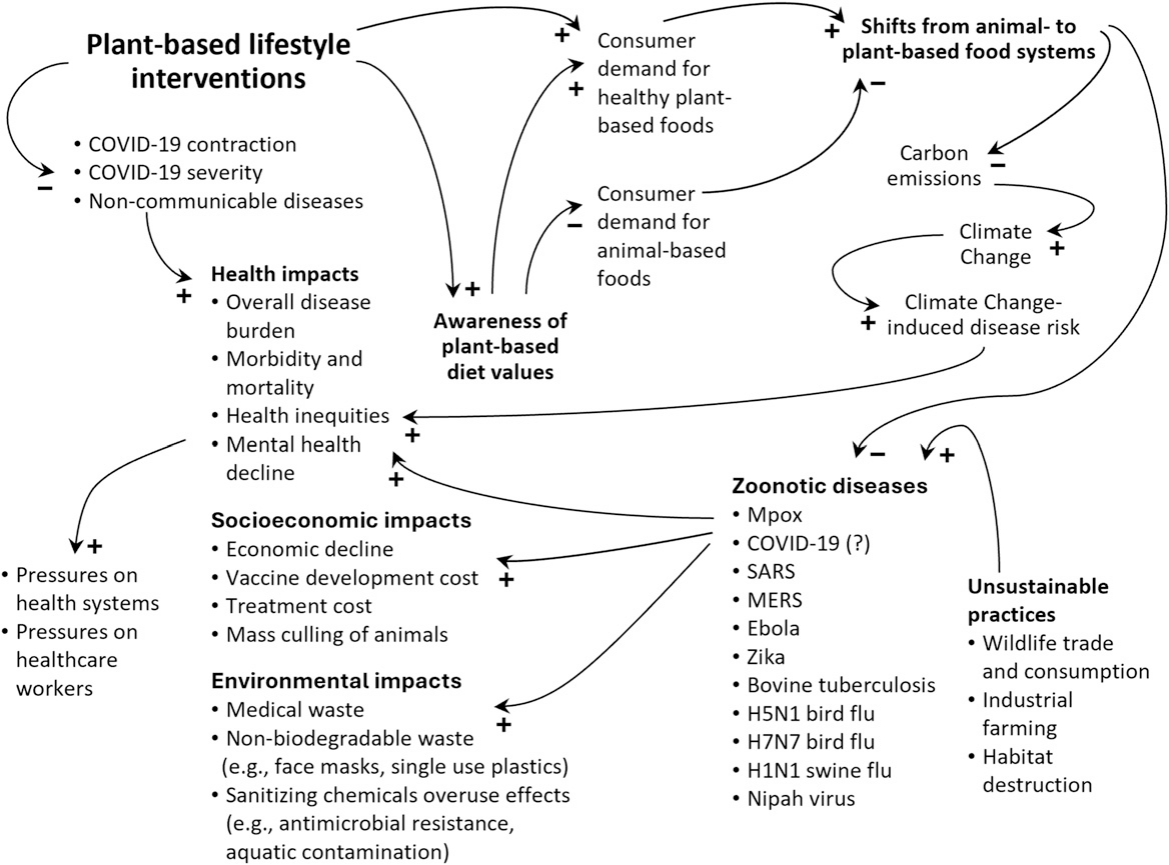
Causal-loop diagram illustrating the burden of zoonoses and the immediate and long-term resolving outcomes that we can expect from plant-based lifestyle interventions. Arrows show cause-and-effect links with tail items affecting head items. A plus sign (+) at the arrowhead suggests a change in the same direction; if the tail item increases, the head item also increases. A minus sign (−) indicates change in the opposite direction; if the tail item increases, the head item decreases.

The COVID-19 pandemic amplified the overall disease burden, led to societal and environmental costs, and added strain on health-care systems. Although limited in number and methodological rigor, observational findings suggest that plant-based diets can potentially lower the risk and severity of COVID-19. Plant-based diets are worthy of consideration in lifestyle medicine, as they may also reduce the risk of obesity, diabetes, cancer, and heart disease, potentially mediating the effect of comorbidity on health outcomes. We provide a conceptual picture of the potential of plant-based lifestyle interventions in Figure 1, using the “causal-loop” modeling concept in systems thinking to illustrate interconnectedness—providing a basis for further research, hypothesis testing, and policy considerations.^56^ As depicted in the figure, a health-sector-driven shift toward plant-based diets could drive consumer demand and encourage a transition from animal to plant-based agriculture, thereby contributing to longer-term disease prevention by mitigating future epidemics and pandemics, while also lowering greenhouse gas emissions that contribute to climate change and consequential ailments.

Hospitals in the US, the UK, and Germany have developed programs to raise awareness of health and environmental benefits of plant-based foods through hospital plant-based menus.^16-19,57^ There is an opportunity to go a step further through enabling health-care policies and budgetary decisions that support plant-based diet prescriptions by medical practitioners and dietitians.

However, several notable obstacles must be overcome for the formal adoption of plant-based lifestyle interventions. Firstly, research on plant-based diets remains limited. Improving the definitional clarity of plant-based diets is necessary, as existing ambiguity makes it difficult to evaluate associated research^12^ and impedes comparative analysis, replicability, and the development of clearly informed plant-based dietary interventions.^11^ Given that all COVID-19 studies examined here were observational, we cannot make a definitive causal inference. While this highlights the need for robust intervention studies that can provide more convincing evidence on the value of plant-based diets for protecting against COVID-19, research limitations alone should not deter a preventive health approach, as the issue is not a lack of evidence but rather that the existing evidence is indicative.

Secondly, there is a need for enhancing physicians’ understanding of plant-based nutrition. As recommended in existing guides, consuming adequate protein and a variety of nutritious plant-based foods is essential to ensure gut microbial diversity and prevent potential nutrient deficiencies (e.g., of iron, zinc, calcium, vitamin D, vitamin B12, and omega-3 fatty acids), particularly for children, older adults, and women during heavy menstruation, and prenatal and postnatal periods.^14,58^ However, physicians may not always be equipped with sufficient nutrition knowledge. For example, in surveys, health-care professionals and medical students indicated inadequate nutrition knowledge though generally aware of the benefits of plant-based diets.^59,60^ Such knowledge gap is concerning, given research which shows that doctors’ recommendations have the highest potential to encourage the adoption of plant-based diets, even among those reluctant to do so.^61^ Formal training to equip practitioners with plant-based nutrition knowledge as well as strategies for communicating with patients would enable them to offer better guidance on healthier choices, prevent diet-related deficiencies, or prescribe plant-based diets to those who may benefit from it.

A third challenge would be ensuring equitable access to affordable, culturally inclusive, nutritionally adequate, and appealing alternatives. Access to affordable and nutritious plant-based food, and the time, equipment and skills required to plan and prepare meals may be a barrier for population groups living in socioeconomic deprivation. These elements suggest the value of collaborations between relevant community groups, food scientists, and local plant-based food producers to enable the development of affordable, healthier alternatives that contain wholefoods. Recommendations by health-care professionals also need to be accompanied by resources to develop plant-based food knowledge and food preparation skills so the transition is to wholefood (e.g., minimally processed nuts, seeds, fruits, vegetables, legumes and grains to help meet protein, iron, zinc and calcium requirements). Whole plant-based foods offer a range of benefits including high micronutrient density, good quality fiber that can support gastrointestinal, cardiovascular, and immune systems, and sources of fats that can decrease caloric density.^14^ Such materials could help raise awareness of the importance of using food labels to make healthy plant-based food choices given the rising availability of ultra-processed meat and dairy alternatives that are counterproductive to the targeted health benefits.

Lastly, success of plant-based lifestyle interventions would depend on overcoming contextual and personal barriers. Practitioner observations in some Western countries suggest that besides inadequate nutrition education, barriers to such interventions include a profit-driven model (i.e., fee-for-service) that incentivizes treatment over prevention (the latter being less profitable as it means reduced patient visits, prescriptions and procedures), disregard of nutrition sciences among physicians, and preservation of the nutrition status quo.^62,63^ Furthermore, patients’ psychological barriers, such as denial, inertia, and cognitive dissonance, also add to the challenge.^63^ A strong meat-eating culture is also often accompanied by an underlying resistance towards vegan and vegetarian diet rationalizations.^64^ Such resistance may be intensified through animal agriculture industry pushback—as we have seen in the long history of counteractions towards civil society-led advocacy for dietary shifts in the US^65^ and efforts to discredit studies such as the 2019 Eat-Lancet report through media framing in the US, the UK, Australia and New Zealand.^66^ Understanding the full extent of such personal and contextual barriers through structured surveys, media studies, and qualitative enquiries and drawing from examples such as the mentioned hospital-based plant-based programmes^16,17,19^ could help guide health policy, education, and communication strategies.

Given the existing albeit limited evidence, we believe plant-based diets offer a potentially effective preventive lifestyle intervention to reduce the burden of disease—in alignment with disease prevention as a primary goal and outcome in health services. While not easy, the approach warrants consideration given its potential for savings in health costs and lives lost, along with contributions to longer-term global outcomes of zoonotic and climate-induced disease prevention that would occur if population-level dietary changes and consumer demand lead to shifts from animal to plant-based agriculture.

## Supplemental Material

Supplemental material - The Potential of Plant-Based Lifestyle Interventions to Reduce the Burden of Disease in a Multi-Crisis Era Supplemental material for The Potential of Plant-Based Lifestyle Interventions to Reduce the Burden of Disease in a Multi-Crisis Era by Komathi Kolandai, Nicholas Wright, Luke Wilson, Heleen Haitjema, Summer Rangimaarie Wright, Meika Foster, George Laking, Marissa Kelaher, Reen Skaria, Jennifer Douglas, Mok Keong Liew, Marion Leighton, Deborah Brunt, Fuchsia Gold-Smith, Mark Craig, Thomas Joseph, Wayne Hurlow, Cheryl Pittar, Sarah Mortimer in American Journal of Lifestyle Medicine.
