# Supplementary material for: The Potential of Plant-Based Lifestyle Interventions to Reduce the Burden of Disease in a Multi-Crisis Era
Source: Am J Lifestyle Med. 2026 Jan 28:15598276261418594. Online ahead of print. doi: 10.1177/15598276261418594 (PMC12851919; doi:10.1177/15598276261418594)
Supplement: Supplemental material - The Potential of Plant-Based Lifestyle Interventions to Reduce the Burden of Disease in a Multi-Crisis Era [file sj-pdf-1-ajl-10.1177_15598276261418594.pdf]

Supplementary Document 1 – Brief narrative review

Article title: The potential of plant-based lifestyle interventions to reduce the burden of disease in a multi-crisis era

Author (Year)	Country	Sample	Design	Summary of findings
Kim et al. (2021)[1]	France, Germany, Italy, Spain, UK, USA	568 (healthcare workers highly exposed to COVID-19)	Case-control	After adjusting for demographics (age, sex, race/ethnicity, county) and other potential confounders (medical specialty, smoking, physical activity), those consuming plant-based diets showed 73% lower odds of severe COVID-19 symptoms.
Hou, Su, and Chao (2022)[2]	Taiwan	509 (patients diagnosed with COVID-19)	Cross-sectional	Analysis of medical records and retrospective diet-data showed that a vegetarian diet was associated with lower COVID-19 severity, particularly among those aged ≥ 65 years after adjusting for potential confounding factors.
Soltanieh et al. (2023)[3]	Iran	141 (treatment-seeking patients)	Cross-sectional	A higher plant-based diet index score was associated with a lower risk of COVID-19 hospitalisation after adjusting for confounders such as age, sex, and body mass index.
Almasi et al. (2025)[4]	Iran	684 (patients who had recovered from COVID-19)	Cross-sectional	Analysis of medical records and follow up interviews showed that a higher plant-based diet index (PDI) score was associated with lower COVID-19 symptoms and duration of disease and hospitalisation. While a healthful PDI score reduced the risk of severe COVID-19, an unhealthful PDI score increased this risk.
Hawryłkowicz et al. (2025)[5]	Poland	550 (patients diagnosed with COVID-19)	Retrospective survey	A higher adherence to a semi-vegetarian dietary pattern was associated with lower odds of COVID-19 hospitalisation and lower disease severity after adjusting for confounders such as smoking status, chronic diseases and intake of vitamin/mineral supplements.
Deschasaux-Tanguy et al. (2021)[6]	France	7,766 (general population)	Prospective cohort	Greater consumption of vitamins C, K (sourced from plant-based foods), and B9, along with increased intake of fruits and vegetables, was linked to a reduced likelihood of experiencing symptomatic COVID-19 infection. This study accounted for potential confounders such as such as demographics, smoking status, co-existing health issues and COVID-19 protective behaviours.
Gołębiowska et al. (2023)[7]	Poland	17,000 (general population)	Cross-sectional	While vegan and vegetarian diets were not associated with COVID-19 outcomes, consuming balanced meals according to the food pyramid decreased the likelihood of COVID-19 infection after controlling for factors such as age, gender, and marital status.
Merino et al. (2021)[8]	UK, USA	592,571 (general population)	Prospective cohort	Consuming healthy plant foods (high healthful PDI score) was associated with lower COVID-19 risk and severity after adjusting for demographics (e.g., age, sex, race), lifestyle factors (e.g., smoking, physical activity), and COVID-19 protective behaviour (mask wearing)
Darand et al. (2024)[9]	Iran	8,157	Cross-sectional	An unhealthful PDI score (reflecting a dietary pattern of less healthy plant foods, such as refined

Author (Year)	Country	Sample	Design	Summary of findings
				grains, sugary drinks, and desserts) was associated with a higher risk of COVID-19 infection after adjusting for confounders such smoking status, physical activity, and chronic disease. Findings suggest the importance of quality in plant-based diets to ensure improvements in population health outcomes.
Acosta-Navarro et al. (2024)[10]	Brazil	702 (general population)	Prospective cohort	Those on plant-based and vegetarian diets had 39% lower odds of COVID-19 infection than those on an omnivorous diet after adjusting for confounders such as body mass index, physical activity, and pre-existing health conditions. However, COVID-19 severity and duration were not significantly associated with diet in this study.
Janko, Haussmann, and Patel (2025)[11]	UK	170 (Seventh-day Adventist Christians)	Longitudinal	Follow-up at 2 years showed borderline significant association (p = 0.05) between vegetarian status and COVID-19 symptom severity. Among those reporting COVID-19 infection at follow-up, ten (33.3%) were on plant-based diets, and twenty (66.7%) were meat eaters.

References

1. Kim H, Rebholz CM, Hegde S, et al. Plant-based diets, pescatarian diets and COVID-19 severity: A population-based case-control study in six countries. *BMJ Nutr Prev Health.* 2021; 4(1):257-266. doi:10.1136/bmjnph-2021-000272

2. Hou Y-C, Su W-L, Chao Y-C. COVID-19 illness severity in the elderly in relation to vegetarian and non-vegetarian diets: A single-center experience. *Front Nutr.* 2022; 9:1-7. doi:10.3389/fnut.2022.837458

3. Soltanieh S, Salavatizadeh M, Ghazanfari T, et al. Plant-based diet and COVID-19 severity: Results from a cross-sectional study. *BMJ Nutr Prev Health.* 2023; 6:182-187. doi:10.1136/bmjnph-2023-000688

4. Almasi F, Nemati M, Izadi N, et al. The Correlation of Plant-Based Diet with COVID-19 Severity and Symptoms among Individuals Recovered from the Disease. *Int J Nutr Sci.* 2025; 10(1):36-49. doi:10.30476/ijns.2024.104955.1377

5. Hawryłkowicz V, Stasiewicz B, Korus S, et al. Associations between dietary patterns and the occurrence of hospitalization and gastrointestinal disorders—A retrospective study of COVID-19 patients. *Nutrients.* 2025; 17(5). doi:10.3390/nu17050800

6. Deschasaux-Tanguy M, Srour B, Bourhis L, et al. Nutritional risk factors for SARS-CoV-2 infection: A prospective study within the NutriNet-Santé cohort. *BMC Med.* 2021; 19(1):290. doi:10.1186/s12916-021-02168-1

7. Gołębiowska J, Zimny-Zając A, Makuch S, et al. The impact of different types of diet on the prevention of diseases among Polish inhabitants, including COVID-19 disease. *Nutrients.* 2023; 15(18). doi:10.3390/nu15183947

8. Merino J, Joshi A, D., Nguyen L, H. , et al. Diet quality and risk and severity of COVID-19: A prospective cohort study. *Gut.* 2021; 70(11):2096-2104. doi:10.1136/gutjnl-2021-325353

9. Darand M, Golpour-Hamedani S, Karimi E, et al. The association between adherence to unhealthy plant-based diet and risk of COVID-19: a cross-sectional study. *BMC Infect Dis.* 2024; 24(1):1245. doi:10.1186/s12879-024-10115-7

10. Acosta-Navarro JC, Dias LF, de Gouveia LAG, et al. Vegetarian and plant-based diets associated with lower incidence of COVID-19. *BMJ Nutr Prev Health.* 2024; 7(1):4-13. doi:10.1136/bmjnph-2023-000629

11. Janko RK, Haussmann I, Patel A. A Longitudinal Investigation of the Prevalence and Incidence of Self-Reported COVID-19 Disease and the Pandemic's Impact Among Seventh-day Adventist and Non-Adventists Living in the UK. *J Relig Health.* 2025; 64(1):685-695. doi:10.1007/s10943-024-02129-x
